# Supplementary figures and images for: An acidic microenvironment in Tuberculosis increases extracellular matrix degradation by regulating macrophage inflammatory responses
Source: PLoS Pathog. 2023 Jul 7;19(7):e1011495. doi: 10.1371/journal.ppat.1011495 (PMC10355421; doi:10.1371/journal.ppat.1011495)

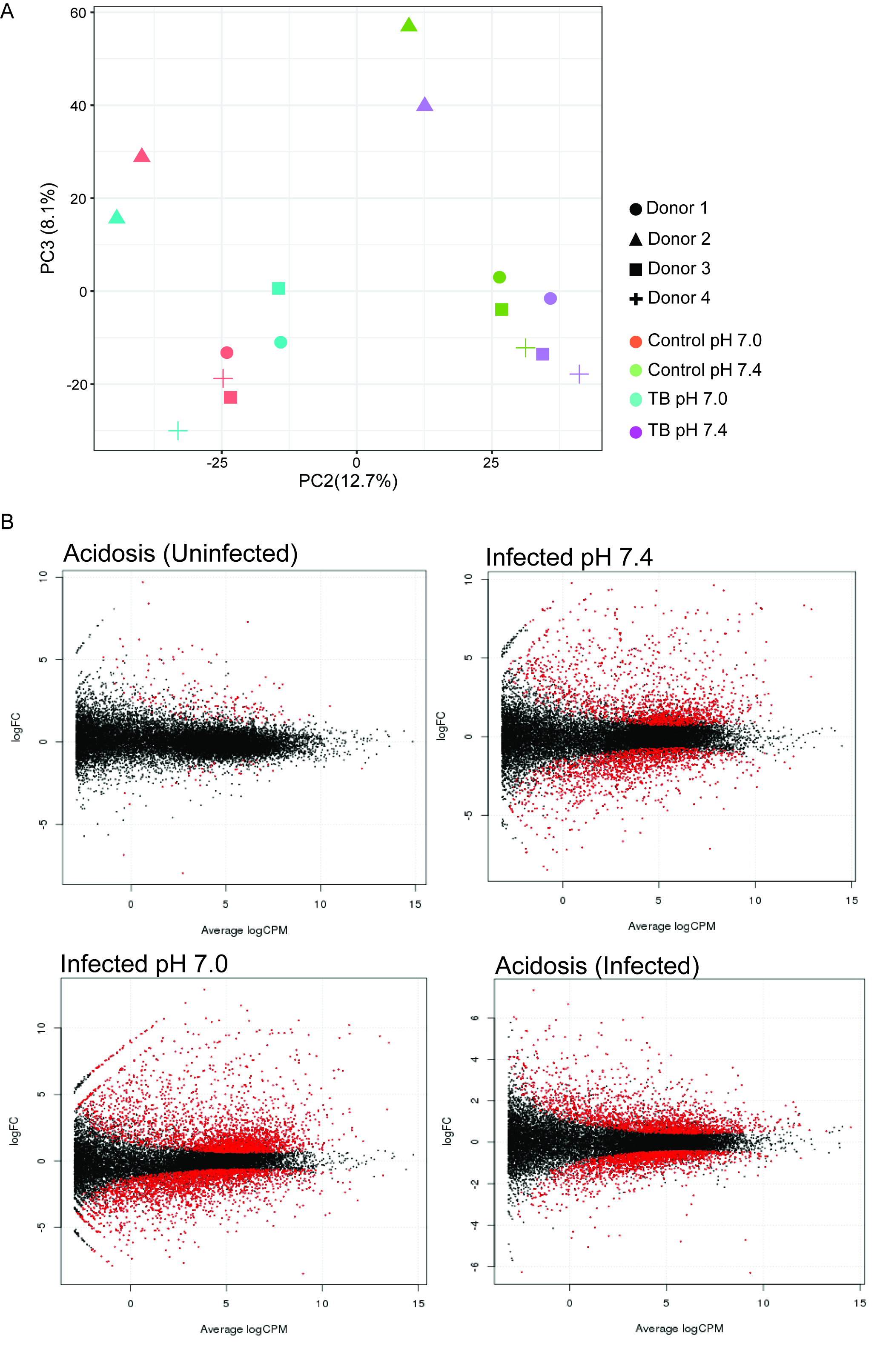

Supplement: S1 Fig — (A) Principal Component Analysis (PCA) showing the second and third principal component on the x and y axis respectively. Total variance attributable to that PC indicated. (B) Smear plots of gene expression in the Effect of Acidosis (uninfected), Effect of Infection at pH 7.4 and pH 7.0, and the Effect of Acidosis (infected) analyses. Log2 Fold Change on the y-axis and Log Counts per Million (CPM) x-axis. Individual genes with FDR<0.01 plotted in red. (TIF) [file ppat.1011495.s001.tif]

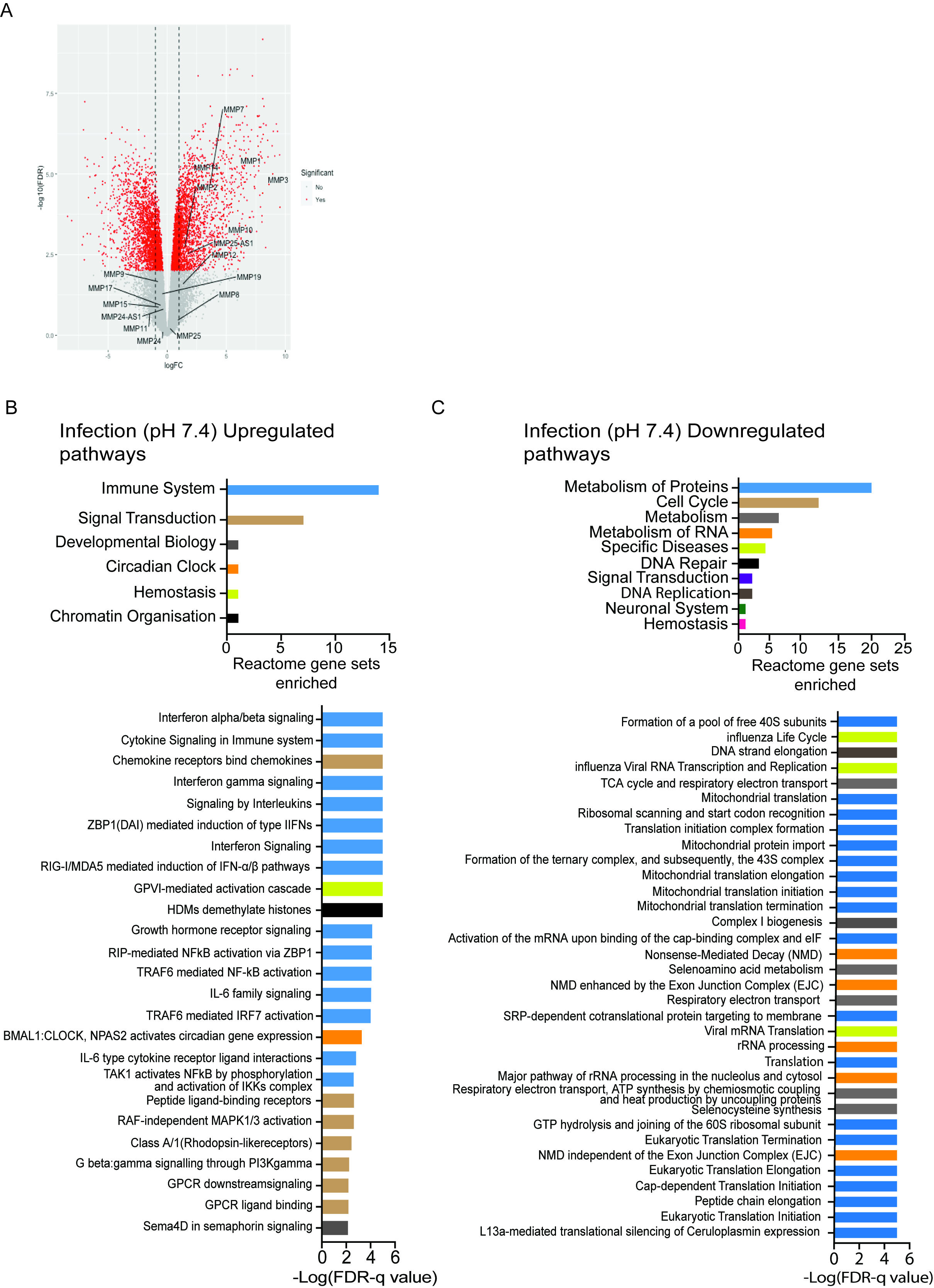

Supplement: S2 Fig — (A) Volcano plot with differential expression results by infection at pH 7.4. MMP genes labelled. Significantly regulated genes marked in red. (B) Reactome Gene sets enriched amongst genes upregulated by M.tb infection of MDMs at pH 7.4. Top panel shows number of enriched gene sets ordered by the uppermost level of the Reactome pathway event hierarchy. Gene sets were considered enriched only if FDR q-value <0.05 when genes were ranked by both fold change and significance (p value) to highlight the most significantly regulated gene sets. Lower panel displays individual gene sets. Plotted FDR-q value is significance ranking value. (C) Downregulated gene sets. (TIF) [file ppat.1011495.s002.tif]

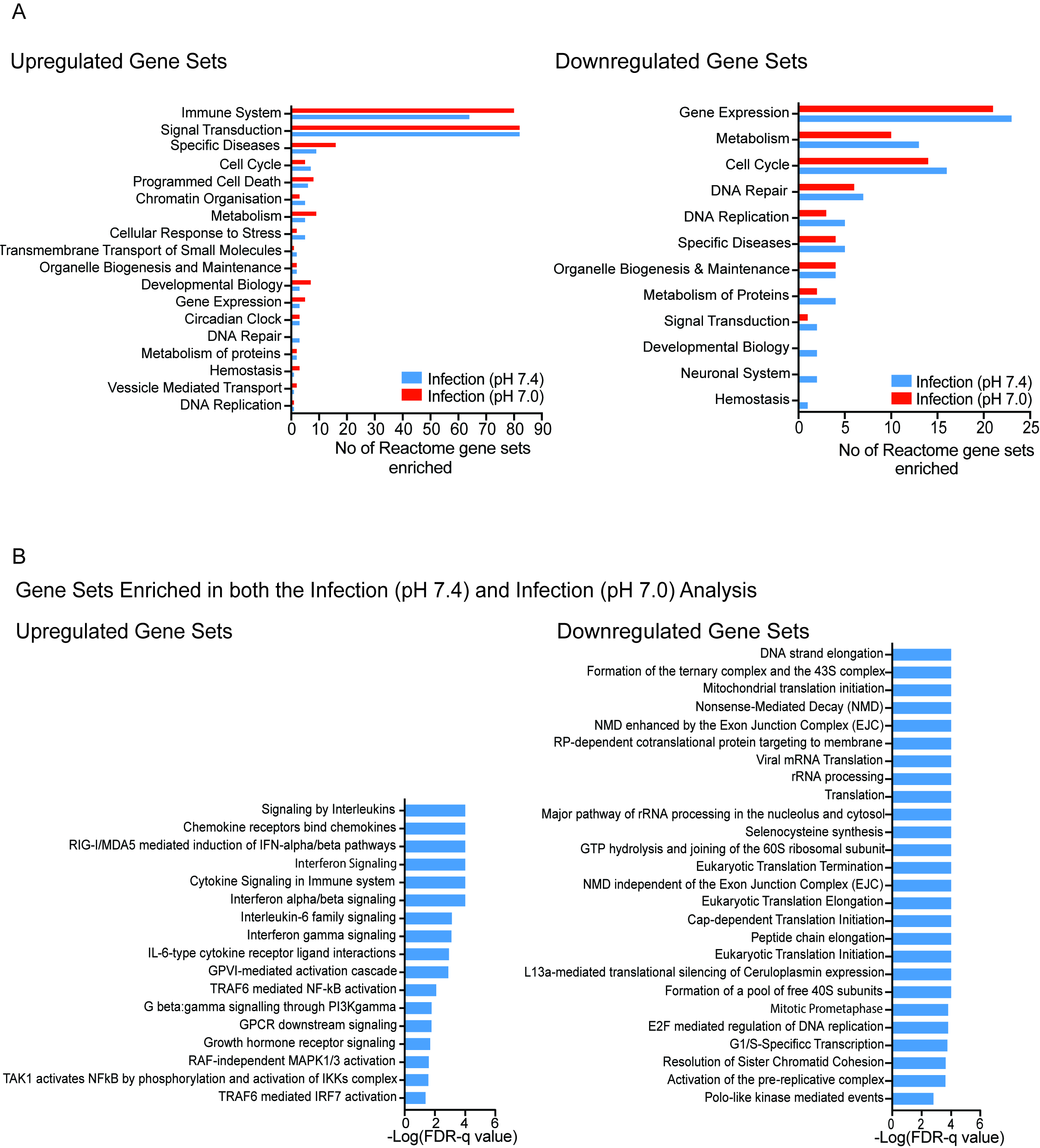

Supplement: S3 Fig — Results of GSEA when genes in the Infected (pH 7.4) and Infected (pH 7.0) analysis are ranked by p value (A) Comparison of the number of gene sets up and down regulated by Infection (pH 7.4) and Infection (pH 7.0) classified by the uppermost level of the Reactome pathway event hierarchy. (B) Gene sets enriched in both the Infection (pH 7.4) and the Infection (pH 7.0) analysis. (TIF) [file ppat.1011495.s003.tif]

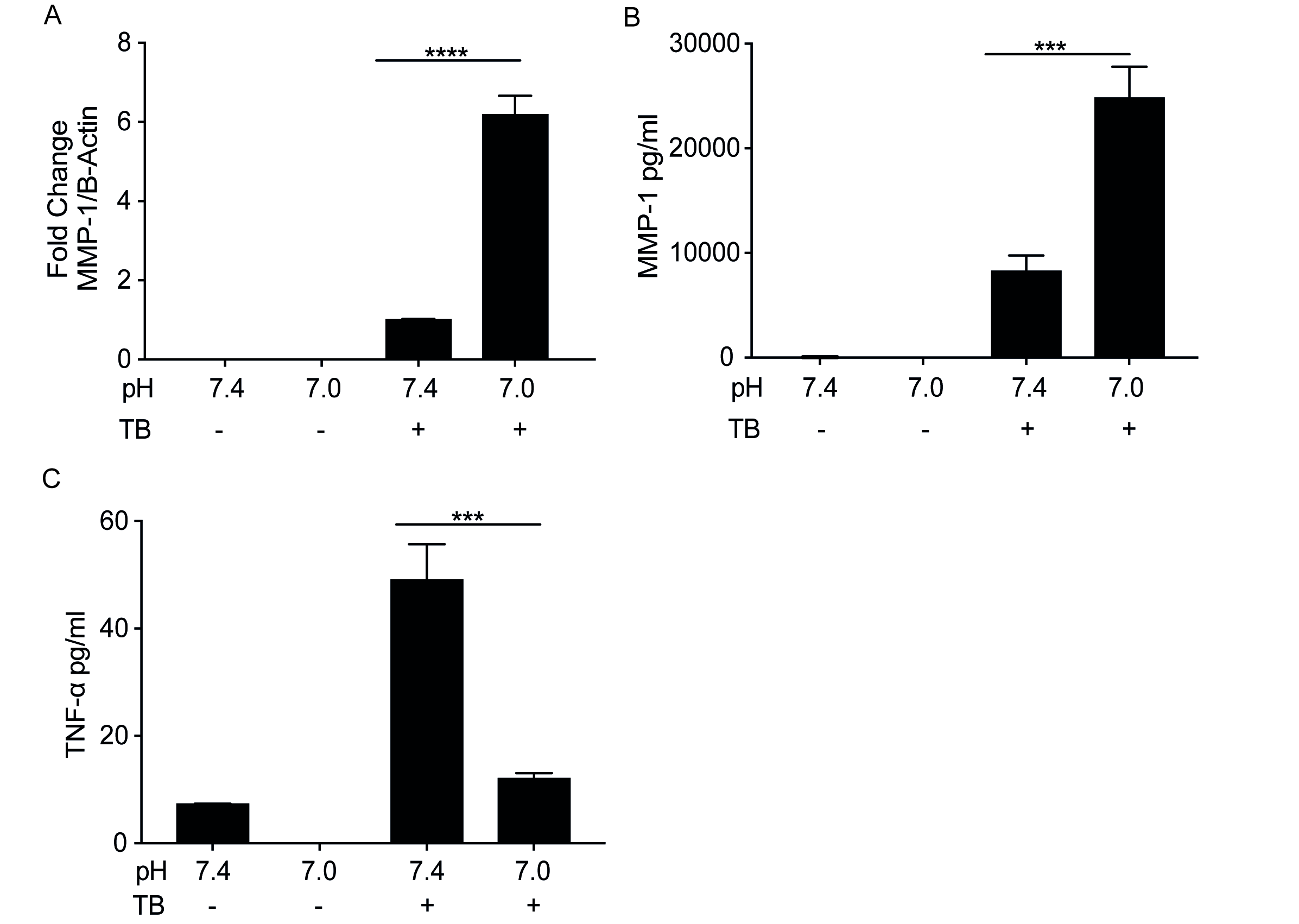

Supplement: S4 Fig — M.tb infected macrophages were cultured as described in the methods section except 10% AB Human serum was used rather than FCS. Graphs show MMP-1 expression (A) and secretion (B) and TNF-α secretion (C) after 24 hours. Graphs are representative of n = 2 donors. Bars are means +/- SD. *p<0.05, **p<0.01, ***p<0.001, ****p<0.0001. NS–not significant. (TIF) [file ppat.1011495.s004.tif]
