# Supplementary material for: An acidic microenvironment in Tuberculosis increases extracellular matrix degradation by regulating macrophage inflammatory responses
Source: PLoS Pathog. 2023 Jul 7;19(7):e1011495. doi: 10.1371/journal.ppat.1011495 (PMC10355421; doi:10.1371/journal.ppat.1011495)
Supplement: Attachment — Submitted filename: Letter responding to reviewers comments.docx [file ppat.1011495.s007.docx]

London North West University NHS Trust

Department of Infectious Diseases

Northwick Park Hospital

Watford Road

Harrow

Middlesex

HA1 3UJ

Dr Ashley Michael Whittington

[A.Whittington@nhs.net](mailto:A.Whittington@nhs.net)

Tel: 07590554725

24^th^ March 2023

Dear Editor,

We would like to thank you for considering our manuscript "An acidic microenvironment in Tuberculosis increases extracellular matrix degradation by regulating macrophage inflammatory responses". In particular we would like to thank the Reviewers for their detailed and thorough review and insightful comments. We have revised the manuscript to take into account the comments from the reviewers. The following is a detailed description of the changes.

- Both Reviewers 1 and 3 expressed concerns that the discussion overstretched data to suggest TDAG-8 could be a target for host directed therapy - “the idea that TDAG-8 itself could be a host-directed therapy for TB patients is a stretch based on the data presented and the fact that blocking this receptor is expected to have many off target effects in the discussion”. Reviewer 3 also expressed concerns that we did not discuss how our conclusions based on a simplified in vitro model may not be directly translatable to patients - “As the authors would surely attest, human TB lesions are far more complex than can be represented by the models presented here”

We thank the reviewers for these comments. We acknowledge that this is very early translational research and more work, including animal model investigation, is required to determine if the upregulation of MMPs and downregulation of macrophage cytokine secretion translates into worsening tissue destruction and inflammation in patients/animals with TB. This work is planned and the hope is that publication of this work will support funding for further study. To address these important concerns the discussion has been revised including specifically paragraph 1 p20, paragraph 2 p 21 and the final paragraph on p 23 where these limitations are discussed.

- Reviewer 3 expressed concern that our use of TB when describing our findings in the discussion was misleading – “The conclusions in the discussion mentioning “TB”. The authors should revise their conclusions to address all mention of the proposed mechanisms’ relevance to “TB”, as this undoubtedly will imply human TB to most readers”. They kindly highlighted several parts of the text they recommended needing rewriting to avoid this including p21 first paragraph and p22 second paragraph.

We understand the importance if this clarification and regret the original text may have been misleading. The parts of the text identified by the reviewer have been extensively rewritten to ensure the reader is clear that our findings were in M.tb infected macrophages *in vitro* and not in patients with TB. For example Page 21 first paragraph now reads “Extracellular acidosis and lactate thus independently enhance secretion of MMP-1 and its activators from M.tb infected macrophages without significant change in inhibiting TIMPs” and concludes “Microenvironment pH and lactate enhancement of macrophage MMP-1 activity may therefore worsen morbidity in TB patients however this requires confirmation in *in vivo* models”. The second paragraph on page 22 now reads “Together these observations show that microenvironment acidosis, signalled via TDAG-8 expression in M.tb infected macrophages suppresses inflammatory, and enhances ECM turnover pathways.”

In addition to the discussion a number of specific points were made:

- We are not told the source of H37Rv? It was grown in broth culture which creates clumps. An MOI of 1 was used. How was this determined and how was clumping assessed and dealt with?

The source of the H37Rv was The National Collection of Type Cultures (UKHSA, UK) and this is now included in the Methods section, first paragraph p24. The optimal method for culturing TB to prevent clumping and the determination of the correct volume of TB needed at a certain optical density to ensure MOI of 1 has been established in our laboratory over a number of years and published on numerous occasions. The first paragraph p24 now includes details of how clumping is prevented - “Clumping was prevented by circular agitation (100rpm) and brief sonication prior to use”. It also includes a reference to an earlier paper (Price NM *et al*) that details how MOI was determined using colony plating of TB from lysed macrophages.

- Macrophages were cultivated in FCS which itself activates human cells. It is important to know whether cells cultivated in human AB serum provide the same result?

We thank the reviewer for suggesting these important experiments. We have performed several of the key experiments again using human AB serum rather than FCS and found identical results – increased MMP-1 gene expression and secretion and decreased TNF-α secretion from M.tb infected macrophages with acidosis. These results have been included as Supplementary Figure 4 and discussed in the main text on p14 end of the first paragraph.

- Macrophages were cultivated in M-CSF which creates one subset of macrophages. What was the rationale for this approach?

We thank the reviewer for raising this important point and apologise it was not clarified in the text. In established infection the majority of M.tb infected cells are macrophages derived from blood born monocyte precursors (Repasy T *et al* Plos Pathogens 2013) hence a desire to study this cell type. Culturing human monocytes using M-CSF alone generates an unpolarised “M0” macrophage with plasticity to develop into either a classically activated (M1) subtype or an alternatively activated (M2) phenotype (Jaguin M *et al* Cell Immunol 2013). As both the stimulus and the microenvironment can cause changes in macrophage polarisation we were keen to study the effect of M.tb infection and acidosis/lactate on unpolarised cells. A line has been added to page 7 paragraph 1 to include this explanation.

- Acidosis is known to affect phagocytosis (and C-type lectins were down). The authors should assess bacterial uptake under the various conditions of their assays.

We agree that acidosis effects on phagocytosis are possible and would be an interesting area of further study. As shown in figure 8D however intracellular bacterial counts were similar after 24 hours of culture regardless of acidosis or lactate concentration. We therefore do not believe that any difference in M.tb uptake with acidosis has impacted the findings of this study significantly and did not pursue this are area further in this work. We have added a line at the end of page 22 paragraph 1 to explain this to the reader.

- The donor and pH grouping and size in Fig. 1A make it difficult to discern the finding of “demonstrated distinct transcription signatures for M.tb infection and acidosis”.

We apologise for the lack of clarity in this figure and we have amended Figure 1A to make the image clearer.

- Fig. 5 D-L is missing 7.0 uninfected in the graphs

This was an error in generating the figures and pH 7.0 controls were used in each experiment. We apologise for this oversight and this has been corrected on a revised Fig 5.

- Although the protein data in Fig. 5 generally recapitulate the transcriptomic data for MMPs, most of the cytokine data were different. No adequate explanation was given. The authors conclude in the discussion that acidosis effects on cytokines is complex. Thus, the significance of the findings is in question.

We agree that the findings that several cytokines, in particular TNF-α, did not show increased secretion despite increases in gene expression was surprising. We discuss in paragraph 2 p21 the possible mechanisms including cytosolic retention and post transcriptional regulation with reference to the work of others who have made similar observations. This paragraph has been rewritten with further explanation and the phrase “acidosis effects on cytokines is complex” has been removed to improve clarity and address the concerns of several of the reviewers as discussed above. We acknowledge that if cytokine gene expression increases are not matched by increases in secretion the interpretation of some of the sequencing data is challenging. The most significantly upregulated pathways with acidosis however related to Extracellular matrix turnover and collagen degradation which forms the bulk of our analysis and discussion and in this area we have shown protein secretion matching the expression data.

- Fig. 6A time point? Fig. 6 J: recovery was modest and from the methods section, the KD efficiency, esp. for OGR-1 was weak. Did the authors optimize the timing of KD for these experiments?

We apologise to the reviewers for not making this clear in the methods. Gene knockdown experiments were optimised for transfection reagent concentration, cell seeding densities and transfection time over numerous experiments. Unfortunately prolonged exposure to the transfection reagent resulted in cytotoxicity and the optimal time for transfection was 8hrs. Text has been added to p28 paragraph 3 to inform the reader.

- Figure 7: Overall cellular architecture is hard to visualize. It is surprising that theTDAG-8 is said to stain strongly in the necrotic center (although staining appears throughout to this reviewer). Necrotic centers usually lack intact macrophages. Is the visualized protein cell-associated in the necrotic center? Why not intense staining in the intermediate zone macrophages surrounding the necrotic center? I am not quite sure what I am looking at with the MGCs? Again, cellular architecture is not clear. 7F would benefit from arrows since I am not sure what we are looking at. Would the tissue cellular architecture information be improved by also staining NHP granulomas which allow for more defined cellular architecture? Several labs have available tissue and are willing to share.

We agree with the reviewers that staining for TDAG-8 was seen throughout the node and not just in the necrotic centre and apologise for not making this clear. We have amended paragraph 2 p14 for clarity - “TDAG-8 stained throughout the lymph node but staining was more marked in the necrotic centre of granulomas where M.tb-infected macrophages reside”. There is some non cell associated staining in the areas of necrosis but there is cell specific staining in the monocytes / MGC as shown in Fig 7 D and E. The wording in paragraph 2 p14 has been amended to specify this. Figure 7E has been magnified to make the cellular architecture clearer. Arrows and further explanation have been added to Figure 7F and the relevant figure legend. These changes will provide the detail and clarity that the reviewer rightly requests.

- Fig. 8. Based on the information, it is not clear what the conclusion is of the independent effects of lactate vs acidosis? Likewise, the MCT data are limited and confusing to interpret relative to the importance of lactate vs acidosis. The discussion mentions that lactate levels above 30mM are above that described in TB but no reference is given, and how and where this is measured is not mentioned. More clear discussion is needed on the overall interpretation of the results and what they mean.

The thank the reviewers for highlighting these issues with the discussion. The wording on paragraph 1 p21 has been changed to reflect these concerns. References have been added to the discussion that report extracellular lactate levels in TB at different sites.

- Reviewer #2:

1. The authors should update their data on the number of annual TB deaths to the most recent report from 2022.
2. Bottom paragraph on p.5, MDMs not yet defined.
3. “Degradation” is misspelled in several Figures.
4. Although the methods indicate that Bonferroni corrections were used for some of the analyses, it is not clear in the results presentation when such corrections for multiple testing were actually employed (e.g., Fig 3).

We thank the reviewer for identifying these issues. 1. The latest Global TB Report from WHO is now referenced and line 2 p4 amended. 2. MDMs is now defined on first use in the second paragraph p5. 3. These spelling errors have been corrected in affected figures (mainly figure 4). 4. Bonferoni corrections were only used for the *in vitro* cellular data. The “Statistical Analysis” section of methods has been amended to clarify this.

We are grateful to the reviewers for their comments and hope that these corrections address the concerns they raised and add the clarity requested for the reader.

Kind Regards,

Dr Ashley Whittington

Consultant in Infectious Diseases
