# Supplementary material for: An acidic microenvironment in Tuberculosis increases extracellular matrix degradation by regulating macrophage inflammatory responses
Source: PLoS Pathog. 2023 Jul 7;19(7):e1011495. doi: 10.1371/journal.ppat.1011495 (PMC10355421; doi:10.1371/journal.ppat.1011495)
Supplement: Attachment — Submitted filename: Letter responding to reviewers comments v2.docx [file ppat.1011495.s008.docx]

London North West University NHS Trust

Department of Infectious Diseases

Northwick Park Hospital

Watford Road

Harrow

Middlesex

HA1 3UJ

Dr Ashley Michael Whittington

[A.Whittington@nhs.net](mailto:A.Whittington@nhs.net)

Tel: 07590554725

19th June 2023

Dear Editor,

We would like to thank you for considering our manuscript "An acidic microenvironment in Tuberculosis increases extracellular matrix degradation by regulating macrophage inflammatory responses".

As requested please find a revised version of the manuscript that includes a figure summarising the key findings in the paper to address concerns from the reviewers.

.

Kind Regards,

Dr Ashley Whittington

Consultant in Infectious Diseases
